# Complete Genome Sequence of *Simiduia agarivorans* SA1**^T^**, a Marine Bacterium Able To Degrade a Variety of Polysaccharides

**DOI:** 10.1128/genomeA.00039-12

**Published:** 2013-01-15

**Authors:** Silk Yu Lin, Wung Yang Shieh, Jwo-Sheng Chen, Sen-Lin Tang

**Affiliations:** a Institute of Oceanography, National Taiwan University, Taipei, Taiwan; b College of Health Care, China Medical University, Taichung, Taiwan; c Biodiversity Research Center, Academia Sinica, Taipei, Taiwan

## Abstract

*Simiduia agarivorans* strain SA1**^T^** is able to degrade a variety of polysaccharides found in marine algae, plants, and animals. The genome of *S. agarivorans* SA1**^T^** consists of a single chromosome (4,309,711 bp), and its information may provide insights into the polysaccharide-degrading capability, cell division, flagellar motility, and chemotaxis of this bacterium.

## GENOME ANNOUNCEMENT

*Simiduia agarivorans* SA1^T^ is a marine bacterium isolated from shallow coastal seawater and is able to degrade a variety of refractory polysaccharides, such as agar, alginate, cellulose, and chitin (1). The phylogeny analysis based on the 16S rRNA gene showed that *Simiduia* species formed a distinct lineage within other bacterial neighbors of the class *Gammaproteobacteria* (1). 

*S. agarivorans* SA1^T^ is a type strain in this clade. Moreover, this bacterium reproduces itself by a unique multiple offspring formation. The finishing and annotation of the *S. agarivorans* SA1^T^ genome are useful to elucidate its genomic features, including those relevant to its versatile metabolic capabilities and cell division manner.

The sequencing technologies used included the Illumina GA2 (Solexa), HiSeq 2000 (Illumina), and 454 GS Junior sequencing (Roche) systems (2). The Illumina paired-end sequencing was performed at the Biodiversity Research Center of Academia Sinica (Taiwan) and yielded ~21,945,490 reads of 63 bp in length. The output data were processed and assembled using Off-Line Basecaller (OLB) software, producing 135 contigs covering approximately 4.2 Mb. The data were combined with the results of another Illumina mate-pair sequencing that was performed at Yourgene BioScience (Taiwan), and 22,750,330 mate-end reads of 40 bp in length were obtained. The data sets were assembled using Velvet software (3). The resulting assembly consisted of 17 large contigs of over 71 kb (maximum contig size of 950 kb). The 454 GS Junior sequencing was performed at the Microarray and Sequencing Core of Academia Sinica, generating 143,752 reads with a modal length of 446 bp. The output data were processed and assembled using Newbler software (4), producing 87 contigs covering approximately 4.3 Mb. After combining all of the data, a genome scaffold of 4.309 Mb was generated. The remaining gaps and unassertive assembled regions were verified by PCR/Applied Biosystems (ABI) sequencing to obtain a single contig.

An optical map of the *S. agarivorans* SA1^T^ genome was constructed at Yourgene BioScience with the HindIII restriction enzyme, yielding 504 ordered restriction fragments. The genome size was estimated to be approximately 4.29 Mb, which was determined from the sum of all restriction fragments of the map. The assembly was finished by scaffolding the contigs on the optical map using the MapSolver software (OpGen). The correct sequence mapping relied upon the optical map. The genome was annotated using the NCBI Prokaryotic Genomes Automatic Annotation Pipeline (PGAAP) employed for submission (http://www.ncbi.nlm.nih.gov/genomes/static/Pipeline.html).

The *S. agarivorans* SA1^T^ genome encodes a 4,309,711-bp circular molecule with a G+C content of 55.9% and no plasmid. We compiled 3,786 (The original gene number is the sum of cds + rRNA + tRNA.) coding sequences (CDSs), 3 rRNA operons, and 41 tRNAs from the nucleotide sequence. The genome contains more than five polysaccharide-hydrolyzing enzyme systems, with a total of 45 CDSs involved in the hydrolysis of agar, alginate, cellulose, chitin, and xylan. It contains more than 47 CDSs involved in the cell division process, including those for cell division regulation, murein and shape determination, chromosome partition, Z-ring formation, the membrane-embedding Tol-Pal system, and amidase.

### Nucleotide sequence accession number. 

The complete genome sequence of *S. agarivorans* SA1^T^ has been deposited in GenBank under accession number CP003746.

## References

[1] Kislyuk AO Katz LS Agrawal S Hagen MS Conley AB Jayaraman P Nelakuditi V Humphrey JC Sammons SA Govil D Mair RD Tatti KM Tondella ML Harcourt BH Mayer LW Jordan IK 2010 A computational genomics pipeline for prokaryotic sequencing projects. Bioinformatics 26:1819–1826 20519285 10.1093/bioinformatics/btq284 PMC2905547

[2] Zerbino DR Birney E 2008 Velvet: algorithms for *de novo* short read assembly using de Bruijn graphs. Genome Res. 18:821–829 18349386 10.1101/gr.074492.107 PMC2336801

[3] Margulies M Egholm M Altman WE Attiya S Bader JS Bemben LA Berka J Braverman MS Chen YJ Chen Z Dewell SB Du L Fierro JM Gomes XV Godwin BC He W Helgesen S Ho CH Ho CH Irzyk GP Jando SC Alenquer ML Jarvie TP Jirage KB Kim JB Knight JR Lanza JR Leamon JH Lefkowitz SM Lei M Li J Lohman KL Lu H Makhijani VB McDade KE McKenna MP Myers EW Nickerson E Nobile JR Plant R Puc BP Ronan MT Roth GT Sarkis GJ Simons JF Simpson JW Srinivasan M Tartaro KR Tomasz A Vogt KA Volkmer GA Wang SH Wang Y Weiner MP Yu P Begley RF Rothberg JM 2005 Genome sequencing in microfabricated high-density picolitre reactors. Nature 437:376–380 16056220 10.1038/nature03959 PMC1464427

[4] Shieh WY Liu TY Lin SY Jean WD Chen JS 2008 *Simiduia agarivorans* gen. nov., sp. nov., a marine, agarolytic bacterium isolated from shallow coastal water from Keelung, Taiwan. Int. J. Syst. Evol. Microbiol. 58:895–900 18398190 10.1099/ijs.0.65371-0

